# Myosin Heavy-Chain Messenger Ribonucleic Acid (mRNA) Expression and Fibre Cross-Sectional Area in Masseter, Digastric, Gastrocnemius and Soleus Muscles of Young and Adult Rats

**DOI:** 10.3390/biology12060842

**Published:** 2023-06-09

**Authors:** Aikaterini Lagou, Leandra Schaub, Aouatef Ait-Lounis, Balazs J. Denes, Stavros Kiliaridis, Gregory S. Antonarakis

**Affiliations:** 1 Division of Orthodontics, University Clinics of Dental Medicine, University of Geneva, 1211 Geneva, Switzerland; 2 Department of Orthodontics and Dentofacial Orthopedics, Dental School, Medical Faculty, University of Bern, 3010 Bern, Switzerland

**Keywords:** masticatory muscles, masticatory function, diet

## Abstract

**Simple Summary:**

The muscles of mastication, providing the forces allowing us to chew our food properly, may be influenced by what we eat as well as growth. The aim of this study was to evaluate the expression of different molecules that contain the instructions to make the proteins necessary for the muscle fibres, as well as the size of the muscle fibres during growth. In order to do this, we used rats that were evaluated at two different ages corresponding to youth and to young adulthood. The size of the muscle fibres increased with age, as well as the type of proteins made, which favoured muscle fibres of the fast-twitch type with a high fatiguability. These types of fibres are built for short, powerful bursts of energy. These results give us insight into the normal development of the muscles used for chewing, which often consists of short, powerful strokes. This knowledge is important as it will allow us to compare different diets to see how diet can influence muscle characteristics, as well as diseases that may affect muscle characteristics.

**Abstract:**

Different demands on the muscles of mastication may influence their functional profile (size and distribution of muscle fibre types), which may change during growth and maturation, potentially influencing craniofacial growth. The aim of this study was to evaluate mRNA expression and cross-sectional area of masticatory muscle fibres compared with limb muscles in young and adult rats. Twenty-four rats were sacrificed at two different ages, namely 12 at 4 weeks (young) and 12 at 26 weeks (adult). The masseter, digastric, gastrocnemius and soleus muscles were dissected. Gene expression of myosin heavy-chain isoforms *Myh7* (MyHC-I), *Myh2* (MyHC-IIa), *Myh4* (MyHC-IIb) and *Myh1* (MyHC-IIx) in the muscles was measured using qRT-PCR RNA analysis, and immunofluorescence staining was performed to measure the cross-sectional area of different muscle fibre types. Different muscle types and ages were compared. Significant differences were found in the functional profile between masticatory and limb muscles. For the masticatory muscles, there was an increase in *Myh4* expression with age, and this change was more intense for the masseter muscles, which also presented an increase in *Myh1* expression, similarly to limb muscles. The fibre cross-sectional area of the masticatory muscles was generally smaller in young rats; however, this difference was less pronounced than in limb muscles.

## 1. Introduction

Masticatory muscle function has been found to potentially influence dentofacial growth and morphology [1,2]. This finding falls within the notion that maxillofacial growth can be regulated by the soft tissues surrounding the orofacial structures, including the masticatory muscles. To evaluate the functional capacity of the masticatory muscles, several non-invasive clinical methods exist, including ultrasonographic measurements of the thickness or volume of the masticatory muscles, maximal molar bite force assessment, or electromyographic recordings of the activity of the masticatory muscles, amongst others. Other thicknesses and volumetric measurements less used include computed tomography (CT) or magnetic resonance imaging (MRI) methods, but these methods involve either irradiation (for CT imaging) or long waiting lists and increased costs (for MRI imaging). An assessment of the composition of the masticatory muscles, however, with regard to the different fibre types and their size, is more difficult in human subjects since a muscle biopsy would be needed, which would not be ethically acceptable except in certain circumstances such as in patients undergoing orthognathic surgery.

In order to be able to assess and measure the composition of the masticatory muscles with regard to the different fibre types and their size, animal models are required. In doing so, it has been shown in experimental studies involving an animal model (commonly using a rat model) that changes in the functional demands of the masticatory muscles lead to muscular adaptation, influencing muscle fibre type composition and distribution, fibre diameter and size, as well as total muscle weight [3,4]. The above characteristics may provide the functional profile of the muscle in mature subjects, which seems to be relatively stable, provided that no functional changes occur [5] and that no pathological disturbances are present [6]. This is the case for adult individuals, although it may not be the case for growing or younger subjects when maturation takes place at different time points in different muscle groups [7]. Thus, during the growth period of the individual, the masticatory muscles possibly develop according to the demands exerted on the different muscle groups depending on their specific function [8].

In mammals, the functional demands of the jaw-opening muscles (e.g., the digastric muscles) may start earlier in life due to the suckling action, compared to the mandible elevator muscles (e.g., the masseter muscles), whose functional demands start after the eruption of the posterior teeth, and more specifically the molars which are primordial for proper mastication [9]. Moreover, since craniofacial structures may differ to a large extent in their pattern and rate of growth, this could perhaps be to some degree dependent on their growth trajectory or their relative maturity. Since craniofacial growth in the rat model seems to follow a maturity gradient that is similar to that observed in humans [10], the use of this model could provide some understanding into the maturation changes occurring in human beings by extrapolation, albeit appreciating that masticatory function between rats and humans differs. In addition, muscle function has been shown to have a direct effect on the craniofacial structures, depending on the anatomical location and growth direction of the muscles in question [11]. A study looking into rat craniofacial growth found that masticatory function tended to have a more important effect on the structures that were the least mature, demonstrating an inter-relationship between masticatory muscle function and growth maturity during craniofacial development [11].

The hypothesis of the present study is, therefore, that the muscle fibre profile of the digastric and masseter muscles does not show the same progress in maturation, and this is also different to that seen in the limb muscles in a rat model. The null hypothesis is that no differences exist between the digastric and masseter muscles with respect to the development of their functional profile when evaluating myosin expression and the cross-sectional area of the muscle fibres.

The aim of the present study was to investigate the changes in the mRNA expression and cross-sectional area of masticatory muscle fibres, compared with limb muscles, in a rat model from early childhood to adulthood in two masticatory muscle groups, each one of them representing a different masticatory functional demand, namely: the masseter muscles representing the mandible elevator muscles; and the digastric muscles representing the jaw-opening muscles.

## 2. Materials and Methods

### 2.1. Ethical Approval

The present study was approved by the ethics committee for animal research (number GE/15/20A). The reporting of the present experiments follows the ARRIVE guidelines (Animal Research: Reporting of In Vivo Experiments) [12].

### 2.2. Subjects

Twenty-four male Wistar rats were used for this study, composed of two different age groups: twelve 4-week-old rats that correspond to the young group and twelve 26-week-old rats that correspond to the adult group.

In order to define the number of animals necessary to be included in the study, the sample size calculation was performed using Stata/IC 16.1, and this calculation was based on the information obtained from Sano et al. (2007) [13], where the size and the cross-sectional area of the masseter and the digastric muscle fibres was described. The standard deviation of the thickness of the muscle fibres varied appreciably (168 to 235 μm^2^) for the type IIA fibres depending on the region of the deep masseter muscle of 10-week-old male rats and was approximately 200 μm^2^ for the type IIB fibres. Thus, the latter was chosen as an average of the expected standard deviation of the sample. The mean fibre size varied notably depending on the region and the fibre type from 938 to 1926 μm^2^. The calculation was based on the assumption that an increase of at least 250 μm^2^ would be detected between the two age groups. A sample size of 24 rats, with n = 12 in the young group and n = 12 in the adult group, was expected to have 80 percent power (power (1 − ß) = 0.8) to detect this 250 μm^2^ difference between the two age groups using the two-sample difference test, with a 0.05 two-sided significance level (alpha (α) = 0.05).

All of the animals were housed, two per cage. All rats spent the first 3 weeks of their life fed by their mother (suckling and weaning phases), and after this point, they were fed an ordinary rat diet in the form of pellets and water ad libitum. The light/dark regime was 12/12 h. After their arrival, all the animals were kept in quarantine for a period of one week. At the age of 4 weeks and 26 weeks, the body weight was measured to ensure their well-being as requested by Swiss animal welfare law (LPA, art 4b). The animals were subsequently sacrificed. Initially, they were anesthetized by inhalation of isoflurane (5%), and while under anaesthesia they received an intraperitoneal injection of pentobarbital (150 mg/kg, diluted to 200 mg/mL). After the rats were sacrificed, three muscle groups were dissected, namely the masseter, digastric and gastrocnemius/soleus (calf) muscles.

### 2.3. Methods

All of the muscle samples were frozen by liquid-nitrogen-cooled isopentane at −140 to −149 °C and stored at −80 °C [14]. The frozen samples were cut in a cryostat in sections of 200 μm for RNA extraction and 10 μm for the immunohistochemical analysis.

#### 2.3.1. RNA Analysis

Total ribonucleic acid (RNA) was extracted using NucleoZOL (Macherey-nagel, Duren, Germany). Complementary deoxyribonucleic acid (cDNA) was synthesized from 1 μg RNA using qScript cDNA SuperMix (QIAGEN, Beverly, MA, USA). Polymerase chain reaction (PCR) was performed using the StepOne and StepOnePlus Real-Time PCR Systems (Applied Biosystems, Waltham, MA, USA) and PowerUp SYBER Green Master Mix (Life Technologies, Carlsbad, CA, USA). To determine the changes in steady state, messenger RNA (mRNA) was quantified relative to a standard curve generated with serial dilutions of a reference cDNA preparation and normalized using Ribosomal Protein Large, PO (RPLPO) mRNA. All experiments were repeated at least two times. Real-time quantitative polymerase chain reaction (RT-qPCR) was used to quantify the mRNA transcripts of the myosin heavy-chain (MyHC) isoforms regardless of the type of fibre in which they are expressed. The abundance of *Myh7* (MyHC-I), *Myh2* (MyHC-IIa), *Myh4* (MyHC-IIb) and *Myh1* (MyHC-IIx) mRNA relative to the house-keeping gene Ribosomal Protein Large, PO (RPLPO) mRNA in the masseter, digastric and gastrocnemius/soleus muscles of young (4 weeks) and adult (26 weeks) rats was calculated. Primers are provided in Table 1.

#### 2.3.2. Immunofluorescence Analysis

Immunofluorescence analysis was performed to identify the four fibre types: I, IIa, IIb and IIx. This method was applied to all three groups of muscles (masseter, digastric and gastrocnemius/soleus muscles), and the cross-sectional area of the muscle fibres was calculated. For each muscle sample, four consecutive histological sections were used for the immunofluorescence analysis [15,16,17], and they were incubated using monoclonal antibodies raised against purified heavy-chain myosin. The antibodies and protocol used are outlined in Table 2 and Table 3.

#### 2.3.3. Morphometric Analysis

The sections were scanned by Axio Scan.Z1 slide scanner. Image acquisition and analysis were performed at the Bioimaging Core Facility, Faculty of Medicine, University of Geneva. Using Matlab combined with QuPath algorithms, the area of interest was identified where further analysis took place. In order to evaluate the samples, twelve Regions of Interest (ROI) were placed on each section in a random fashion along the complete available area. In each ROI, an average of 22 fibres were measured, meaning that on each section, an average of 264 fibres were measured. All ROIs were verified for the correct tracing of the fibres, and the number and size of all fibres were measured automatically, excluding the fibres touching the border of the ROI. The number of ROIs was determined by running pilot experiments which showed that with twelve ROIs, the results reach saturation, meaning that increasing the number of ROIs to more than twelve did not lead to any significant changes in the results.

#### 2.3.4. Statistical Analysis

The results of the RNA analysis were tested using two-tailed unpaired Student’s *t*-tests to detect differences between the young and adult rats. The unpaired Student’s *t*-test was also used to test the differences between the size of the muscle fibre types in the two age groups. As far as the differences in size between the muscle groups are concerned, due to the number of muscle groups being three, a one-way analysis of variance (ANOVA) test was performed, followed by post-hoc analysis in the cases where the results indicated statistically significant differences. The statistical analysis of the RNA data was performed in GraphPad PRISM (version 9.3.0 463) software, whereas the muscle size data were analysed using Octave software (version 8.1.0). A *p* value threshold of <0.01 was considered as statistically significant.

## 3. Results

### 3.1. Body Weight

The mean body weight for the young rats was 106 ± 7 g, and for the adult rats was 525 ± 63 g.

### 3.2. RNA Analysis

#### 3.2.1. Masseter Muscle

**Figure 1:**
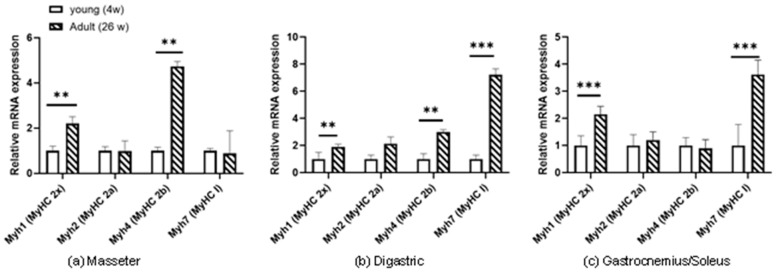
*Myh1, Myh2, Myh4, Myh7* mRNA levels were quantified in young (4 week) and adult (26 week) rats: (**a**) masseter, (**b**) digastric and (**c**) gastrocnemius/soleus muscles. The mRNA transcripts of the MyHC isoforms regardless of the type of fibre in which they are expressed; *Myh7* (MyHC-I); *Myh2* (MyHC-IIa); *Myh4* (MyHC-IIb); *Myh1* (MyHC-IIx). The results were normalized using RPLPO mRNA and are expressed relative to young masseter muscle. **: statistically significant (*p* < 0.01) difference between young and adult rats at the fibres of this muscle ***: statistically significant (*p* < 0.001) difference between young and adult rats at the fibres of this muscle.

In the masseter muscle, *Myh1* (MyHC-IIx) mRNA levels were increased two-fold in the adult compared to the young rat group (*p* < 0.01). Similarly, a significant difference (*p* < 0.01) was measured in the mRNA expression of *Myh4* (MyHC-IIb) fibres in the adult compared to the young rat group, where the adults showed more than a four-fold increase of *Myh4* (MyHC-IIb) mRNA abundance. No change was observed for *Myh2* (MyHC-IIa) and *Myh7* (MyHC-I) mRNA expression between the young and the adult rats (Figure 1a).

#### 3.2.2. Digastric Muscle

In digastric muscle fibres, the *Myh4* (MyHC-IIb) mRNA expression had a three-fold increase (*p* < 0.01), the *Myh1* (MyHC-IIx) mRNA expression had a two-fold increase (*p* < 0.01) and the *Myh7* (MyHC-I) mRNA expression a seven-fold increase (*p* < 0.001) in the adult compared to the young rat group. The *Myh2* (MyHC-IIa) showed no difference between the young and the adult rat group (Figure 1b).

#### 3.2.3. Gastrocnemius/Soleus Muscles

As far as the gastrocnemius/soleus muscle fibres are concerned, the largest and most statistically significant difference was observed between the adult and the young rats for *Myh7* (MyHC-I) mRNA expression that exceeded 3.5 times in the adult compared to the young rat group (*p* < 0.001). The *Myh1* (MyHC-IIx) mRNA expression was also increased two-fold in the adult compared to the young rat group (*p* < 0.001). No difference was observed in the expression of *Myh4* (MyHC-IIb) and *Myh2* (MyHC-IIa) between the adult and the young rat group (Figure 1c).

### 3.3. Morphometric Analysis

**Figure 2:**
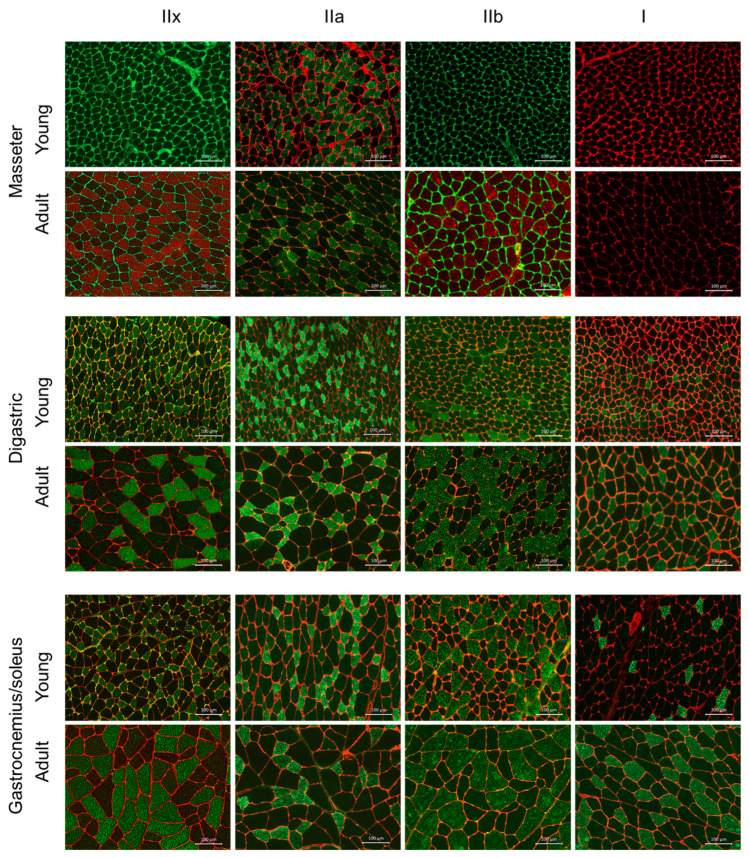
Fibre types in the three different muscles studied (masseter, digastric, gastrocnemius/soleus) in the young and adult rats revealed by immunohistochemical staining. The bottom right-hand corner scale line indicates the length of 100 μm.

The four fibre types were visible for all three muscle groups in adult animals, as well as the digastric and gastrocnemius/soleus muscles in the young animals (Figure 2). However, for the masseter muscle in the young animals, only the type IIa fibres were clearly identified. Therefore, the measurements of the fibre cross-sectional area were performed accordingly.

When the fibre size of the three muscle groups between the young and the adult animals was compared, all fibre types except the type I fibres of the digastric muscle presented a statistically significant difference, the fibres of the adult animals being bigger in size than those of the young animals. In particular, for the masseter muscle, the measurements indicate a two-fold increase in the size of type IIa fibres for the adult rat group. In the case of the digastric muscle, the difference between the young and the adult animal group indicated an almost 2.5- to three-fold increase for the type IIx and IIb fibres, while a smaller increase (1.5-fold) was observed for the type IIa fibres. In the gastrocnemius/soleus muscles, there was also a large 3–3.5-fold increase in all fibre muscle sizes (Table 4).

Concerning the different muscle groups, no statistically significant difference was found between the fibre size measured in the masseter muscles and the digastric muscles, while all of the muscle fibre sizes in the gastrocnemius/soleus muscles were bigger than those measured in the masticatory muscles, both among the young animals as well as the adult animals (Table 4 and Table 5).

## 4. Discussion

The results of the present study show that the functional profile of the masticatory muscles studied underwent maturation changes from a young age to adulthood. These changes were manifested as an increase in mRNA expression of *Myh4* (MyHC-IIb) and *Myh1* (MyHC-IIx) fibres in the masseter muscle, *Myh4* (MyHC-IIb), *Myh1* (MyHC-IIx) and *Myh7* (MyHC-I) fibres in the digastric muscle and *Myh1* (MyHC-IIx) and *Myh7* (MyHC-I) fibres in the gastrocnemius and soleus muscles. As far as the cross-sectional fibre area is concerned, adult rat fibres were clearly larger in size for all fibre types and muscles studied, except for the type I fibres in the digastric muscle.

As previous studies have shown, in the gastrocnemius and soleus muscles, the adult phenotype is determined by an increase in slow-type fibres, which is related to a mature pattern of innervation and physical activity of limb muscles [18] and is in line with the findings of the current study where an increased *Myh7* (MyHC-I) expression was observed in the adult rats compared to the young rats. Similarly, at the level of the masticatory muscles, Odman et al. [3] found that the alteration of the diet from a soft diet to a hard diet (masticatory functional rehabilitation) could trigger a transition of the gene expression isoforms with *Myh7* levels increasing as an adaptation to the increased mechanical load.

The results of the present study also found that there was an increase of the *Myh4* (MyHC-IIb) isoforms in both the masseter and the digastric muscles of adult rats, which could be potentially explained by their increased functional needs due to growth. Kawai et al. [19] found that the anterior belly of the digastric muscle, in a rat model, contained a high percentage of slow-type fibres (type I fibres and hybrid fibres) as this muscle is involved mainly in low-amplitude activities, which are in line with what was found in the present experimental study in the adult rat group.

Concerning the masseter muscles of the young rat group, only type IIa fibres were identified in the present study, which is in agreement with Sano et al. [13], that reported the dominance of type IIa and type IIx fibres in 10-week-old rats, while type I fibres were missing. Furthermore, they measured type IIb fibres to be the largest in size in this muscle, which matches the current observation for the adult rat group. The transition from suckling to mastication has been reported [20,21] to be correlated with an increase in the presence of fast fibres, which is in line with the present observations concerning the mRNA expression of masseter *Myh4* (MyHC-IIb) and *Myh1* (MyHC-IIx) isoforms that increased two-fold to more than four-fold from young to adult rats.

Results from an experimental rat model of artificially-induced oral breathing have shown that oral breathing may affect the morphology and contractile characteristics of the masseter muscles of growing rats, with a smaller cross-sectional area of the fibres and changes in the MyHC composition of these muscles in the growing oral-breathing mice [22,23]. This can support the notion that differences in occlusal characteristics, which can be related to functional demands via the type of breathing in the aforementioned model, can influence fibre size and phenotypes.

These results confirm the present hypothesis that the development of occlusion can influence the maturation of the masticatory muscles. As previously shown by Denes et al. [24], the first molar in the rat model used erupts on average on day 17, the second molar on day 20, and the third molar on day 33. That means that there is a progressive functional demand for the masseter muscle for chewing, which expresses the type IIb isoforms to a greater extent during growth. On the other hand, the functional demands of the digastric muscle start earlier in life, playing an important role in fast movements during the suckling phase. Subsequent to the weaning phase, the role of the digastric muscle changes to a pattern where less fast movement activity is necessary, as this muscle is used during the opening of the mouth before the animal bites onto the pellets.

It is possible that animals in the weaning phase could present larger differences than those observed in the present study. Concerning the limb muscle, the increase of type I fibres could be associated with the increase in the body weight of the rat and the need to manage this weight, as previously observed also for the soleus rat muscle [25].

One limitation of the present study, when compared with other studies [13,17], is that the spatial variability in muscle fibre composition was not addressed, and this could explain some differences between the fibre cross-sectional area measured in this study compared to the results of previous studies. This limitation was addressed by standardizing the muscle sampling procedure and the choice of ROI placement in the histological sections. A further limitation is that the immunofluorescence analysis was not used to evaluate the myosin isoform expression at the protein level, but the aim of the present study was to evaluate this at the level of mRNA.

Another interesting aspect not looked into in the present study is that apart from the effect of age, sex may also influence muscle fibre size, as the sex hormones such as testosterone have the potential to impact the transition from the slower to the faster fibre types during their growth [26]. In the present study, however, all animals were male Wistar rats, which did not allow for an evaluation of the possible role of sex in the maturation process of the masticatory muscles.

While the present study looked at the functional profile of the masticatory muscles through growth and maturation (from a young age to adulthood), no attempt was made to look into age-related changes in these muscles. Previous research has shown that the expression of MyHC mRNA obtained from masseter muscles of rats at 6, 12, 18 and 24 months of age did not show marked age-related changes [27]. The authors of that study concluded that the biological ageing process, from adult to middle age, did not affect the contractile properties of these muscles. A further study looking into age-related changes in the masseter muscles in a rat model also found no changes in fibre type or fibre size with increasing age [28].

The present results might give some insight into the functional changes that may occur in the masticatory muscles in humans, as it has been shown that muscle hypofunction could cause alterations in craniofacial morphology [29]. At the same time, certain types of malocclusions, for example, the anterior open bite, could influence the functional profile of the masticatory muscles. It was observed that masseter muscle characteristics following orthognathic surgery and an improvement of the occlusion could cause changes in the muscle fibre distribution, decreasing the percentage of type I to type II fibres [30,31]. It is also interesting to mention that type II fibres have been found to be more frequently present in individuals with short face patterns, while open bite patients present fewer type II fibres [32]. Nevertheless, it is perhaps too simplistic to extrapolate observations in the rat masticatory muscles to the human masticatory system, and one must interpret results originating from a rat model with caution.

Finally, the results of the current study could also be important in order to understand the normal fibre growth profile of the masticatory musculature, which can give further insight into understanding the potential alterations occurring during growth in relation to pathological factors such as masseter muscle hypertrophy [33].

## 5. Conclusions

- There is a difference in the functional profile between the masticatory and the limb muscles.
- For the masseter and digastric muscles, there is an increase in *Myh4* (MyHC-IIb) expression between young and adult rats, and this change is more intense for the masseter muscles. Both the masseter and the digastric muscles also present an increase in *Myh1* (MyHC-IIx) expression from young to adult rats, similarly to the limb muscles.
- For the masticatory muscles, the fibre cross-sectional area is generally smaller in the young rats compared to the adult rats; however, this difference is less pronounced than in the limb muscles.

## Figures and Tables

**Table 1:** Primers used for Real-Time quantitative polymerase chain reaction.

Gene Symbol (Organism)	Official Name	Sequences
RPLPO (Rat)	Ribosomal Protein Large, PO	Forward CCTTCTCCTTCGGGCTGATC Reverse GGGCTGTAGATGCTGCCATT
Myh1 (Rat)	Rattus norvegicus myosin heavy chain 1	Forward AAGTTCCGCAAGATCCAGCA Reverse TGGATCGATCACTCTTCGCT
Myh2 (Rat)	Rattus norvegicus myosin heavy chain 2	Forward CCAATGAAACCAAGACACCTG Reverse ATCTCTGCTTGAAGTCTGCG
Myh4 (Rat)	Rattus norvegicus myosin heavy chain 4	Forward CGGGTTGAAGACTCTGGCTT Reverse GCTGACACGGTCTGGAAAGA
Myh7 (Rat)	Rattus norvegicus myosin heavy chain 7	Forward AGAGGAGAGGGCGGACATTG Reverse GGCATCCTTAGGGTTGGGTAG

**Table 2:** Protocol used on rat muscle sections for the immunofluorescence staining.

Overview of Immunofluorescence MHC Staining Protocol	Time
Cut O.C.T.-embedded muscle into 10 µm cross-sections and store at −80 °C	
Air dry sections (entire procedure performed at room temperature)	30 min
Block with 2% BSA in PBS	60 min
Apply 1° antibody	60 min (IIa, IIx); Overnight (IIb, I)
PBS wash	3 × 5 min
Apply 2° antibody	60 min
PBS wash	3 × 5 min
Mount coverslips with DAKO vectashield H-1000	

**Table 3:** Antibodies used on rat muscle sections to identify the type I, IIa, IIb and IIx fibres.

Fibres	Primary Antibody and Concentrations	Incubation Time	Secondary Antibody and Concentration (Dilution)
Fibre Type I	BA-D5	overnight	Goat anti-mouse IgG2b_,_ Alexa Fluor 488 (1:500)
Fibre Type IIa	SC-71 (1:100)	1 h	Goat anti-mouse IgG_1,_ Alexa Fluor 488 (1:500)
Fibre Type IIb	BF-F3 (1:100)	overnight	Goat anti-mouse IgM, Alexa Fluor 555 (1:500)
Fibre Type IIx	6H1 (1:10)	1 h	Goat anti-mouse IgM Alexa Fluor 555 (1:500)
Laminin	Anti-Laminin	1 h	Goat anti-rabbit IgG, Alexa Fluor 488 or Goat anti-rabbit IgG, Alexa Fluor 555 (1:500)

**Table 4:** Fibre cross-sectional area (μm^2^) of masseter, digastric and gastrocnemius/soleus muscles.

	Masseter		Digastric		Gastrocnemius/Soleus	
Young	Mean	SD	Mean	SD	Mean	SD
I	-	-	541	40	707 **	71
IIA	573 **	48	549 ***	41	735 ***	132
IIB	-	-	740 ***	177	1275 ***	302
IIX	-	-	557 ***	48	833 ***	161
**Adult**	**mean**	**SD**	**mean**	**SD**	**mean**	**SD**
I	722	300	634	89	2318 **	759
IIA	1086 **	352	818 ***	99	2079 ***	841
IIB	1596	771	2114 ***	363	4212 ***	906
IIX	1373	248	1375 ***	333	3096 ***	632

**: statistically significant (*p* < 0.01) difference between young and adult rats at the fibres of this muscle. ***: statistically significant (*p* < 0.001) difference between young and adult rats at the fibres of this muscle.

**Table 5:** One-way analysis of variance of the fibre cross-sectional area (μm^2^) between masseter, digastric and gastrocnemius/soleus muscles for young and adult rats in all fibre types.

Young	ANOVA Prob > F	ANOVA Post-Hoc *p*-Values		
		Masseter- Digastric	Masseter- Gastrocnemius/Soleus	Digastric- Gastrocnemius/Soleus
I	0.027			0.027
IIA	<0.001	0.808	0.001 **	<0.001 ***
IIB	<0.001			<0.001 ***
IIX	<0.001			<0.001 ***
**Adult**				
I	<0.001	0.980	0.015	<0.001 ***
IIA	0.002	0.661	0.012	0.002 **
IIB	<0.001	0.441	<0.001 ***	<0.001 ***
IIX	<0.001	1.000	<0.001 ***	<0.001 ***

**: statistically significant (*p* < 0.01) difference between the two muscle types of this fibre type. ***: statistically significant (*p* < 0.001) difference between the two muscle types of this fibre type.

## Data Availability

MATLAB codes were developed in-house at the Bioimaging Core Facility, Faculty of Medicine, University of Geneva. The MATLAB code used for tissue alignment and the QuPath code used for morphometric analysis are available at https://gitlab.com/unige_orthodontie/morphometric-analysis/ (accessed on 1 March 2023).
